# Spontaneous Activity in Primary Visual Cortex Relates to Visual Creativity

**DOI:** 10.3389/fnhum.2021.625888

**Published:** 2021-04-01

**Authors:** Yibo Wang, Junchao Li, Zengjian Wang, Bishan Liang, Bingqing Jiao, Peng Zhang, Yingying Huang, Hui Yang, Rengui Yu, Sifang Yu, Delong Zhang, Ming Liu

**Affiliations:** ^1^Guangdong Key Laboratory of Mental Health and Cognitive Science, Center for Studies of Psychological Application, School of Psychology, South China Normal University, Guangzhou, China; ^2^College of Education, Guangdong Polytechnic Normal University, Guangzhou, China

**Keywords:** visual creativity, primary visual area, spontaneous activity, functional brain network, critical time point analysis

## Abstract

Cognitive and neural processes underlying visual creativity have attracted substantial attention. The current research uses a critical time point analysis (CTPA) to examine how spontaneous activity in the primary visual area (PVA) is related to visual creativity. We acquired the functional magnetic resonance imaging (fMRI) data of 16 participants at the resting state and during performing a visual creative synthesis task. According to the CTPA, we then classified spontaneous activity in the PVA into critical time points (CTPs), which reflect the most useful and important functional meaning of the entire resting-state condition, and the remaining time points (RTPs). We constructed functional brain networks based on the brain activity at two different time points and then subsequently based on the brain activity at the task state in a separate manner. We explore the relationship between resting-state and task-fMRI (T-fMRI) functional brain networks. Our results found that: (1) the pattern of spontaneous activity in the PVA may associate with mental imagery, which plays an important role in visual creativity; (2) in comparison with the RTPs-based brain network, the CTP-network showed an increase in global efficiency and a decrease in local efficiency; (3) the regional integrated properties of the CTP-network could predict the integrated properties of the creative-network while the RTP-network could not. Thus, our findings indicated that spontaneous activity in the PVA at CTPs was associated with a visual creative task-evoked brain response. Our findings may provide an insight into how the visual cortex is related to visual creativity.

## Introduction

Visual creativity is an ability of generating useful and novel products in visual forms, which is very useful in the field of drawing, photography, sculpture, and architecture (Aziz-Zadeh et al., 2013). Being a highly complex cognitive function, visual creativity involved multiple processes, such as the generation of ideas *via* spontaneous thinking and the evaluation of ideas, to check whether a new association could be executed or not (Pidgeon et al., 2016; Kleinmintz et al., 2019). Thus, rather than the isolated brain regions, the distributed networks of brain regions are thought to be necessary for visual creativity (Dietrich and Kanso, 2010). Aziz-Zadeh et al. (2013) found that the visual creative synthesis task, in comparison with the mental rotation task, evoked a more robust activity in the premotor cortex, medial prefrontal cortex (PFC), posterior parietal cortex, and dorsolateral PFC. These regions could be divided into different subnetworks, such as the sensorimotor network, default mode network (DMN), and fronto-parietal network (FPN). Previous studies on visual creativity tended to emphasize the role of the DMN and FPN from a perspective of large-scale network interactions (Beaty et al., 2015, 2016). For instance, a study showed that there was a stronger functional connectivity (FC) between the DMN and FPN in the planning of a visual artwork compared to the pure resting mental activities (De Pisapia et al., 2016). Similarly, Zhu et al. (2017) reported that visual creativity was negatively related to FC within the precuneus of the posterior DMN and right middle frontal gyrus (MFG) of the FPN. Together, these studies revealed how the DMN and FPN cooperate to support creative cognition.

However, emerging evidence has indicated that the visual cortex (network) also played an important role in visual creativity. For instance, several studies on task-state neuroimaging showed that the visual cortex had higher responses in creative visual tasks than in uncreative visual tasks (Aziz-Zadeh et al., 2013; Huang et al., 2013; Park et al., 2015). In addition, Chen et al. (2019) reported that visual creativity showed a better performance, which was associated with a stronger resting-state FC between the visual network, DMN, and FPN. Similarly, a study from our laboratory also found that the visual cortex was involved in the prediction of the visual creative performance by using a graph-based resting-state network analysis (Jiao et al., 2017). Nevertheless, a very few works have directly explored the relationship between the visual cortex (network) and visual creativity. Visual creativity is typically defined as breaking of the imagined combinations of familiar patterns and reconstructing practical and original patterns (Finke, 2014). Thus, mental imagery plays a crucial role in visual creativity (Palmiero et al., 2011). Previous studies showed that the visual creative synthesis task was widely used to explore visual creativity, and the generation and manipulation of mental imagery is a key component of it (Finke and Slayton, 1988; Boccia et al., 2015; Palmiero et al., 2015). In addition, spontaneous activity in the primary visual area (PVA), a core region of the visual network, was proved to be tightly associated with mental imagery (Zhang et al., 2018). Taken together, we inferred that there was a relationship between spontaneous activity in the PVA and a neural response to the visual creative synthesis task.

In the resting-state neuroimaging studies, the traditional FC analysis primarily focused on the correlation between the brain regions based on an analysis of the whole scan session. Such an analysis could not evaluate the characteristics of spontaneous activity within a specific brain region (Wang et al., 2008) or capture the potential variation of the brain response over time (Liu and Duyn, 2013). However, the information regarding a specific brain region is stored at a few critical time points (CTPs), which show observable variations in terms of the signal-to-noise ratio (Tagliazucchi et al., 2012). Compared with the traditional FC analysis, the critical time point analysis (CTPA) could examine the brain activities from the CTPs to reflect the most useful and important functional meaning of the entire resting-state condition (Wang et al., 2008). Consequently, the present study using the PVA as a region of interest (ROI) and in combination with the CTPA approach to explore the relationship between spontaneous activity in the visual cortex and a creative task-evoked brain activity. To conclude, we first collected the functional magnetic resonance imaging (fMRI) data from the same sample at rest and during performing a visual creative synthesis task. Then, we used the CTPA to identify the pattern of spontaneous activity in the PVA. Lastly, we constructed both the resting-state and task-state brain network and investigated the relationship between topological attributes of the resting-state and task-fMRI (T-fMRI) brain network.

## Materials and Methods

### Participants

A total of 16 healthy right-hand undergraduates (eight females, mean age of all participants, 21 ± 1.37 years) were recruited from South China Normal University (SCNU) in Guangzhou, China. All participants had a normal or corrected-to-normal vision. None of the participants had a history of neuropsychiatric disorders or previously received creativity training according to their self-report. A written informed consent was obtained from each participant before the study. Our study was approved by the Institutional Review Boards at SCNU. All participants were requested to participate in MRI scans.

### Stimuli and Tasks

**Figure 1:**
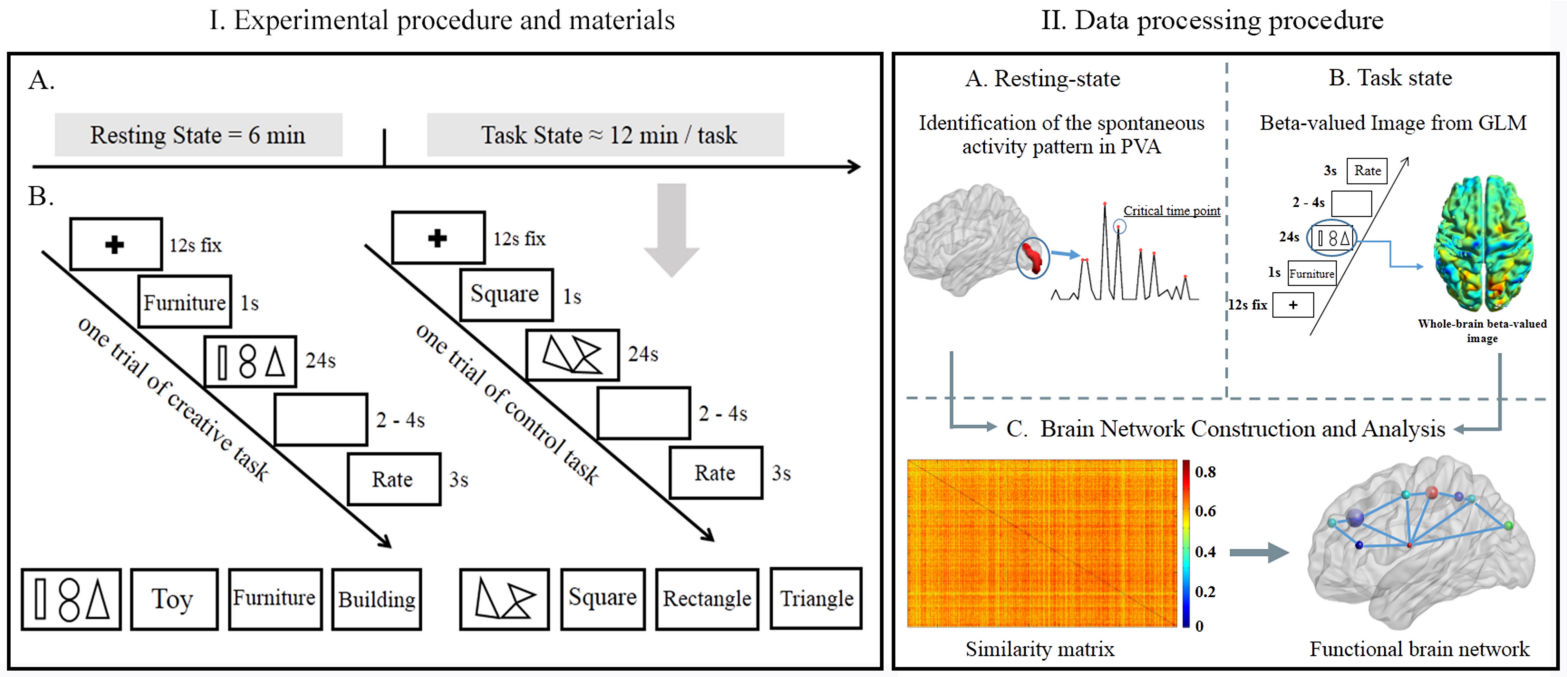
**(I)** Experimental procedure and materials. (A) General experimental procedures in detail; (B) samples of experimental categories and materials in the visual creative synthesis task (left) and control task (right). **(II)** Data processing procedure. From (A to C) represent the resting-state fMRI (R-fMRI) data processing procedure; from (B to C) demonstrate the Task-fMRI (T-fMRI) data processing procedure. PVA, primary visual area; GLM, general linear model.

General experimental procedures are shown in Figure 1IA and the materials used in the visual creative synthesis task and control task (mental rotation task) are shown in Figure 1IB.

In the visual creative synthesis task, 18 pictures were used as stimuli (Aziz-Zadeh et al., 2013; Finke, 2014). Each picture included three line-shaped items, such as triangle, double circle, and rectangle (Figure 1IB). Participants were informed to create a novel and useful product based on the pre-presented three items. This product should belong to one of the six categories, including office appliances, furniture, toys, sport objects, accessories, and buildings. For example, the category clue is furniture, then three simple shapes (rectangle, double circle, and triangle) were presented for 24 s, and the subjects were asked to use these shapes to create a novel product in their mind during this period. After 2–4 s blank interval, participants needed to evaluate the novelty and usefulness of their products by pressing the buttons in the MRI scanner, 1–4 score represented from the lowest novelty or usefulness to highest (“1” and “2” with left hand and “3” and “4” with right hand, Figure 1IB). Each category was shown thrice, but the stimulus items in the picture were different for each time.

In the control task (mental rotation task), the clues were rectangle, triangle, or square, and the stimulus pictures were synthesized by decomposing the regular shape (such as square, triangle, and rectangle) to three parts and rotating an angle with one fixed peak point of each part (Aziz-Zadeh et al., 2013). Participants were asked to reunion the three parts back to a regular shape by using the mental rotation for 24 s and estimated whether these decomposed shapes could be synthesized to the original clue shape. If participants estimated the transformed shape being the same as the shape of the clue, then used the left hand to press the “1” button. Otherwise, they used the right hand to press the “4” button (Figure 1IB). Each regular shape was transformed into six irregular shapes, three for the consistent condition and the remaining three for the inconsistent condition. So, we had 18 transformed pictures in the control task. Consistent and inconsistent trials were balanced in the control task. All stimulus materials were in black and white, at a size of 680 × 400 pixels.

In both the tasks, the participants laid supine on the scanner bed and viewed the visual stimulus back-projected from a screen through a mirror attached on the head coil. E-prime 2.0 was used to present the stimulus (Psychology Software Tools, Inc., Pittsburgh, PA, USA) and the order of stimuli was randomly chosen. The order of two tasks was counterbalanced across the subjects. Before the fMRI, we asked all subjects to perform practice trials on a laptop for being familiar with the instruction of experiment and for understanding the detailed task. After the fMRI, participants needed to perform a recall test to describe the details of products that they created in the scanner for each creative task trial. The recall test can help us confirm whether the subject had completed the task as required.

Before our experiment, 40 students were recruited to evaluate the difficulty and completion time of two tasks. The results showed that there was no significant difference in the completion time and difficulty between the creative task (*M* = 18 s, SD = 3.75) and control task (*M* = 17 s, SD = 3.26), *p* = 0.22.

### MRI Acquisition

All the MRI data were acquired on a 3 T Siemens Trio MR scanner with a 12-channel phased-array head coil at the Brain Imaging Center of SCNU. For each subject, the resting-state fMRI (R-fMRI) data were collected by using an echo planar imaging (EPI) sequence with the following parameters: repetition time (TR) = 2,000 ms, echo time (TE) = 30 ms, flip angle = 90°, field of view (FOV) = 224 mm × 224 mm, in-plane matrix size = 64 × 64, slice thickness = 3.5 mm, voxel size = 3.5 mm × 3.5 mm × 3.5 mm, and 32 axial slices. During the R-fMRI scan, the participants were asked to open their eyes to fix a black cross on the screen and lay in the scanner in a supine position. About 180 functional volumes were obtained in a 6 min scan. The T-fMRI data were collected by using the same sequence with the R-fMRI scan. During the T-fMRI scan, the participants were asked to perform a visual creative synthesis task and a control task. Each run included 18 trials and lasted for 770 s (385 TRs; Figure 1IA). Individual high-resolution brain structural images were acquired by using a three dimensional (3D) T1-weighted magnetization-prepared rapid gradient-echo (MP-RAGE) sequence.

### fMRI Data Preprocessing

We used Statistical Parametric Mapping (SPM8, http://www.fil.ioin.ucl.ac.uk/spm) and Data Processing Assistant for Resting-State fMRI (DPARSF toolkit, http://restingfmri.sourceforge.net) to preprocess the R-fMRI with the following steps: (1) the first 10 volumes of the functional images were discarded to account for signal equilibrium and a participant's adaptation to the immediate environment. (2) Slice timing and head motion correction were applied to the R-fMRI images, and no participant was removed because of a large head motion (larger than 1.5 mm of displacement or 1.5° of rotation in any direction). (3) All images were normalized to the standard Montreal Neurological Institute (MNI) space (voxel size = 3 mm × 3 mm × 3 mm) by using an EPI template and smoothed with a 4 mm full-width at half-maximum (FHWM) Gaussian kernel. (4) The data were band-pass filtered (0.01–0.08 Hz) and detrended to decrease the effects of physiological noise, data drift, and linear trend. (5) We used a linear regression to remove nuisance covariates, including the Friston 24 head motion parameters obtained by a rigid body correction for head motion (Friston et al., 1996), white matter, and cerebrospinal fluid signals.

Preprocessing of the T-fMRI data was performed by using the SPM8. We discarded the first five volumes to account for the signal equilibrium and participant adaptation to the immediate environment. The preprocessing steps of the remaining 380 images included slice timing, head motion correction, the normalization of all images to the MNI space (voxel size = 3 mm × 3 mm × 3 mm) using an EPI template, and smoothing of all images using a 4 mm FHWM Gaussian kernel. High-pass filtering (1/128 s) data were used to rule out the effects of scanner drift.

### Definition of ROI

We chose the PVA (BA17) from the Brodmann's area (BA) atlas as a ROI using the WFU_Pick-Atlas (http://fmri.wfubmc.edu/software/PickAtlas). We normalized the ROI image to the standard EPI template and resampled it to coincide with the preprocessed fMRI images in terms of the voxel size (3 mm × 3 mm × 3 mm). There were 213 voxels in the ROI of the PVA.

### Identification of the Pattern of Spontaneous Activity in the PVA

In this study, we used a CTPA applied by Wang et al. (2008) to identify the pattern of spontaneous activity in the PVA at the resting state. To guarantee the independence of each time point, we chose 34 time points from 170 time points with an interval of 5 TR (i.e., the 1st, 6th, …, 166th time point). For the 34 time points, the following steps were performed on the ROI of each participant based on the height and spatial extent of signal changes:

1. We extracted the blood oxygen level-dependent (BOLD) values from all voxels in the PVA at each time point and transformed them into *Z* values.
2. We calculated the number of voxels with *Z* > 2 at each time point, then ranked these time points in a descending order according to the number of voxels with *Z* > 2.
3. If the number of the voxels with *Z* > 2 in the top 2 time points was both over the 2.5% of the total voxel number in the PVA (i.e., exceeded the Gaussian assumptions), we defined them as CTPs.
4. Other time points were also defined as CTPs, provided that the number of voxels of *Z* > 2 at this time point exceeded 2.5% of the total voxel number within the PVA, and at this time point more than 30% of voxels with *Z* > 2 appeared also in the set of voxels with *Z* > 2 at higher ranked CTPs.

At last, we classified the 34 time points into two classes for each subject: the CTPs and the remaining time points (RTPs) that did not meet the abovementioned conditions. In addition, to exclude the possibility that the movement of the subject in the scanning session might influence the pattern of spontaneous activity in the PVA that we detected, we applied a data exclusion criterion: the parameters of head motion were larger than 1.5° in any of the pitch, yaw, roll angular rotation axes or 1.5 mm in any of *x, y, z* translation direction axes.

To identify a neural network related to spontaneous activity in the PVA, the following procedures were also carried out by using SPM8. For each subject, the image of both CTPs and RTPs was entered into a two-sample *t*-test to detect whether there was a significant difference in the BOLD responses. Then, we obtained an effect of interest (CTPs vs. RTPs) on a voxel-to-voxel basis over the entire brain across all participants. Next, to detect the brain regions related to the PVA-related spontaneous activity at the group level, a set of contrast images were entered into a random effect by using a one-sample two-tailed *t*-test.

### Resting-State-Based Functional Networks

For R-fMRI, a functional network was constructed for each CTP and RTP (Figure 1IIA–C). The nodes were defined by using voxel cubes (a cube consisting of 3 × 3 × 3 voxels), which parceled the whole brain based on the Anatomical Automatic Labeling 90 (AAL90). The edges of the network represented the similarity of BOLD values between every two nodes. By using a cube similarity approach (Tijms et al., 2012), the correlation coefficient of the cubes on the BOLD values in the voxels of defined nodes was measured. Because the cubes with zero variance in BOLD values were excluded (average across all participants, 0.01%), only positive similarity values survived this threshold. After constructing a brain network on each CTP or RTP, we, respectively, averaged all network matrices under CTP or RTP. Finally, we got a mean CTP functional network (CTP-network) and mean RTP functional network (RTP-network) for each participant.

### Task State-Based Functional Networks

After preprocessing for T-fMRI, we performed a general linear model (GLM) analysis to estimate the beta-valued images across the whole brain for each of the 18 trials. For each subject, we obtained the beta brain image, respectively, from the creative manipulation phase and mental rotation phase (duration: 24 s). Using the beta brain images, we could find more accurately the whole brain responses corresponding to a creative processing and control processing. Then, we used the beta brain image of creative task and control task in a separate manner to construct a brain network at an individual subject level using the same procedure as in the R-fMRI data. In the end, we obtained a mean functional brain network under the creative task (creative-network) and control task (control-network, Figure 1IIB,C).

### Network Analysis

We analyzed topological properties of four types of brain networks (CTP-, RTP-, creative-, and control-network) based on a graph theory and used the global efficiency (*E*_glob_) and local efficiency (*E*_loc_) to depict the global network attributes in detail. *E*_glob_ reflects the efficiency of a parallel information transfer among nodes in the whole network, and *E*_loc_ is the average value of the local efficiency of all nodes in the brain and indicates the efficiency of information transfer in the local network and the ability of the network to defend against random attacks. Two parameters were used to describe the local network topological organization: nodal global efficiency (*E*_glob−nodal_) and nodal local efficiency (*E*_loc−nodal_), which together indicated the efficiency of information transfer of a node.

In addition, two normalized global parameters were used to quantify small-world topography. The parameters are normalized clustering coefficient, ⋎ = *C*_*p*_^real^/*C*_*p*_^rand^, and normalized shortest path length, λ = *L*_*p*_^real^/*L*_*p*_^rand^. *C*_*p*_^real^ and *L*_*p*_^real^, which indicated the clustering coefficient and shortest path length of the real brain network in a separate manner. Moreover, *C*_*p*_^rand^ and *L*_*p*_^rand^ represented the mean value of the corresponding parameter derived from 100 random networks that have the same number of edges, nodes, and the distribution of degrees as a real brain network. If a network met the following criteria: ⋎ >> 1 and λ ≈ 1 or σ = ⋎ / λ > 1, we defined it as a small-world network (Watts and Strogatz, 1998).

In the present study, five sparsities (ranging from 0.05 to 0.25 with an interval of 0.05) were used to threshold all elements of each similarity matrix repeatedly, and each sparsity was computed by dividing the maximum possible number of edges in a network by a total number of edges. In the present study, we not only calculated each network parameter that corresponded to respective sparsity but also computed the integrated parameters over the whole range of sparsity values. We defined the integrated global parameters as:

(1) $$\begin{array}{r} {\text{Integrated }X}_{\;}=\;\overset{5}{\underset{K=1}{\sum }}X\left(K\Delta S\right)\Delta S \end{array}$$

Δ*S* means the sparsity interval of 0.05, and *X (K*Δ*S)* represents one of the global network parameters (*E*_glob_ or *E*_loc_) at a sparsity of *K*Δ*S*. In addition, the integrated nodal network parameters are defined as:

(2) $$\begin{array}{r} {\text{Integrated }Y}_{\;}=\;\overset{5}{\underset{K=1}{\sum }}Y\left(i,K\Delta S\right)\Delta S \end{array}$$

*Y (i, K*Δ*S)* indicates one of two nodal parameters (*E*_glob−nodal_ or *E*_loc−nodal_) at a sparsity of *K*Δ*S*.

### Statistical Analysis

Non-parametric permutation tests (Bullmore et al., 1999) were used to detect the difference in the global network parameters between the CTP-network and RTP-network as well as between the creative-network and control-network. The permutation tests were performed 10,000 times on each parameter and got an empirical distribution of the between condition differences and used the 95% distribution as the critical value for a two-tailed test of the null hypothesis with 5% type I error (false positive). Age and gender were regressed out as covariates when we performed the permutation test because these factors would affect the results of the analysis.

Multiple linear regression analysis (MLRA) was used to determine whether the nodal network properties based on spontaneous activity in the PVA could explain the individual variations in the network properties of the creative task across the subjects. Dependent variables were the integrated global network properties (*E*_loc_ or *E*_glob_) of the creative-network, and independent variables were the integrated nodal network properties (*E*_loc−nodal_ or *E*_glob−nodal_) under the CTPs. To reduce the data dimensions, we only used the independent variables that were significantly correlated (Pearson correlation, *p* < 0.01) with the dependent variables in the regression. After obtaining CTP-network-based regress models, which could explain the variations in properties of the creative-network, we tested whether they could be applied to predict the differences in global network properties of the control-network. Besides, we extracted the value of the nodal parameter (*E*_loc−nodal_ or *E*_glob−nodal_) of the RTP-network from the same node used in a CTP-network-based regress model to detect whether the same model obtained from the RTP-network could explain the variance in global network properties of the creative-network.

## Results

### Spontaneous Activity Patterns in PVA

**Figure 2:**
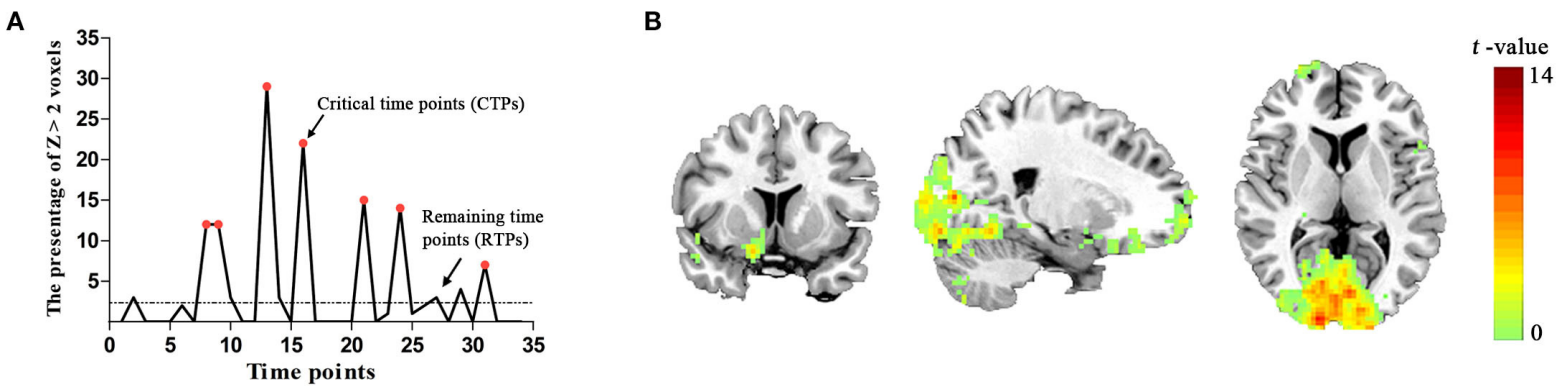
**(A)** The spontaneous brain activity pattern from a participant. The ordinate shows the percentage of *Z* > 2 voxels across the 34 time points. The red points indicate the critical time points (CTPs), and the rest were remaining time points (RTPs). **(B)** Schematic representation of the brain regions representing significant associations with spontaneous activity in the PVA [*p* < 0.05, cluster size > 50 voxels, corrected False Discovery Rate (FDR)].

No participant was excluded for an apparent head motion (i.e., > 1.5° or 1.5 mm) at the resting state. For each subject, we found that the level of brain activity at CTPs was higher than that at RTPs in PVA. Sample data were selected from a single subject (Figure 2A). In the group level, the number (mean ± SD) of CTPs was 6.88 ± 1.63, which exceeded 20% of the 34 time points. In addition, there were 213 voxels in the identified PVA. In our study, the observed number (mean ± SD) of voxels with *Z* > 2 in the PVA at CTPs was 20.19 ± 4.31 (about 9.5% ± 2.0% of the total voxels in ROI), which exceeded the expectation level (2.5%) and suggested that the higher level brain activities in CTPs were correlated with a brain mechanism.

In the group level, we identified that the activity patterns (CTPs > RTPs) of some brain regions were similar to the patterns of spontaneous activity in the PVA. The regions associated with spontaneous activity in the PVA comprised three parts: (1) the bilateral visual areas including the calcarine, lingual gyrus (LING), middle occipital gyrus (MOG), and cuneus (BA 17/18/19), which also extended to the fusiform gyrus (BA 20); (2) the PFC including the bilateral MFG/inferior frontal triangular part (BA 6/8) and right middle frontal orbital part (MFGorb), superior frontal orbital part, superior frontal gyrus (BA 10/11); (3) sensorimotor areas, such as the left precentral gyrus (BA 4). More detailed information are shown in Table 1 and Figure 2B.

**Table 1:** Foci of brain areas located in the neural network associated with the resting-state primary visual area- (PVA-) related spontaneous activity.

**Brain regions**	**Hem**	**CS**	**BA**	**(*x, y, z*)**	***t*-value**
Calcarine/Lingual gyrus/Middle occipital gyrus/ Cuneus/Fusiform gyrus	Bi	4,039	17/18/19	−6, −99, −6	13.43
Middle frontal orbital part/Superior frontal orbital part/Superior frontal gyrus	R	275	10/11	21, 66, −6	5.86
Middle frontal gyrus/Inferior frontal triangular part/Precentral gyrus	L	145	4/6/8/9	−42, 15, 30	5.89
Middle frontal gyrus/ Inferior frontal triangular part	R	106	6/8/45	51, 21 45	5.79

*Hem, hemisphere; CS, cluster size; BA, Brodmann's area; (x, y, z), coordinates of primary peak locations in the Montreal Neurological Institution space; Bi, bilateral hemisphere; R, the right hemisphere; L, the left hemisphere. p < 0.05, cluster size > 50 voxels, FDR-corrected*.

### Comparative Analysis of Network Parameters

**Figure 3:**
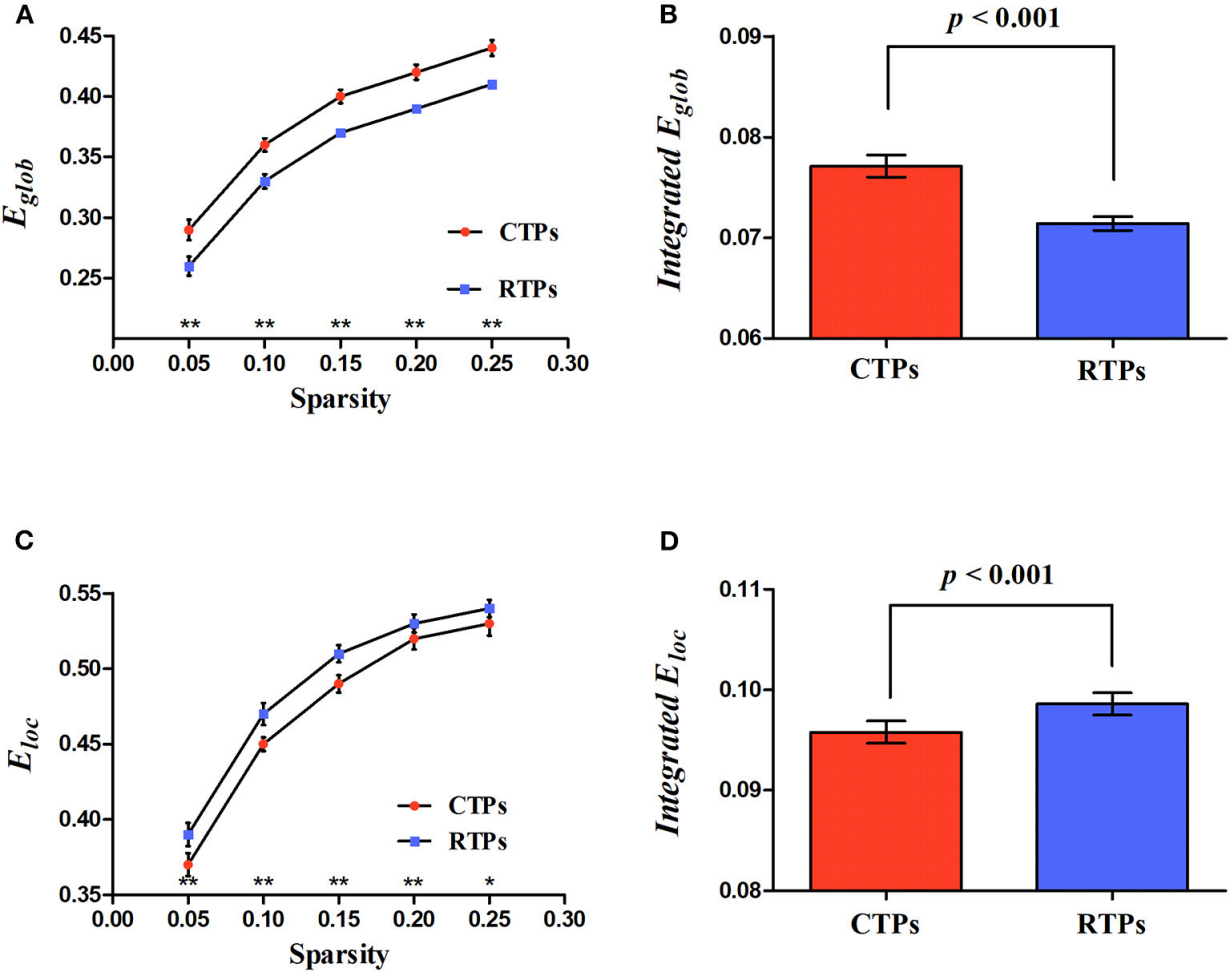
Illustration for the global parameters changing with sparsity **(A,C)** and significant differences in integrated network parameters **(B,D)** at resting state. *E*_glob_, global efficiency; *E*_loc_, local efficiency. The asterisk (* and **) indicates a significant between group difference at *p* < 0.05 and *p* < 0.01.

Four kinds of functional brain networks (CTP-, RTP-, creative-, and control-network) all met the criteria defining small-world networks (i.e., ⋎ >> 1 and λ ≈ 1 or σ = ⋎ / λ >1). Besides, we found that, compared with the RTP-network, the CTP-network showed a significant increase in the integrated global efficiency (*p* < 0.001; Figure 3B) and a decrease in the integrated local efficiency (*p* < 0.001; Figure 3D). There was a significant decrease in the local efficiency in specific sparsity levels (all *p* < 0.050; Figure 3C) and increase in the global efficiency (all *p* < 0.010; Figure 3A) in each sparsity level when comparing the CTP-network with the RTP-network. However, the non-parametric analysis showed that there was no significant difference in the global (all *p* > 0.050) or local efficiency (all *p* > 0.050) between the creative-network and control-network.

### Relationships Between the Integrated Regional Network Parameters of Resting-State and Integrated Global Network Parameters of Task State

**Figure 4:**
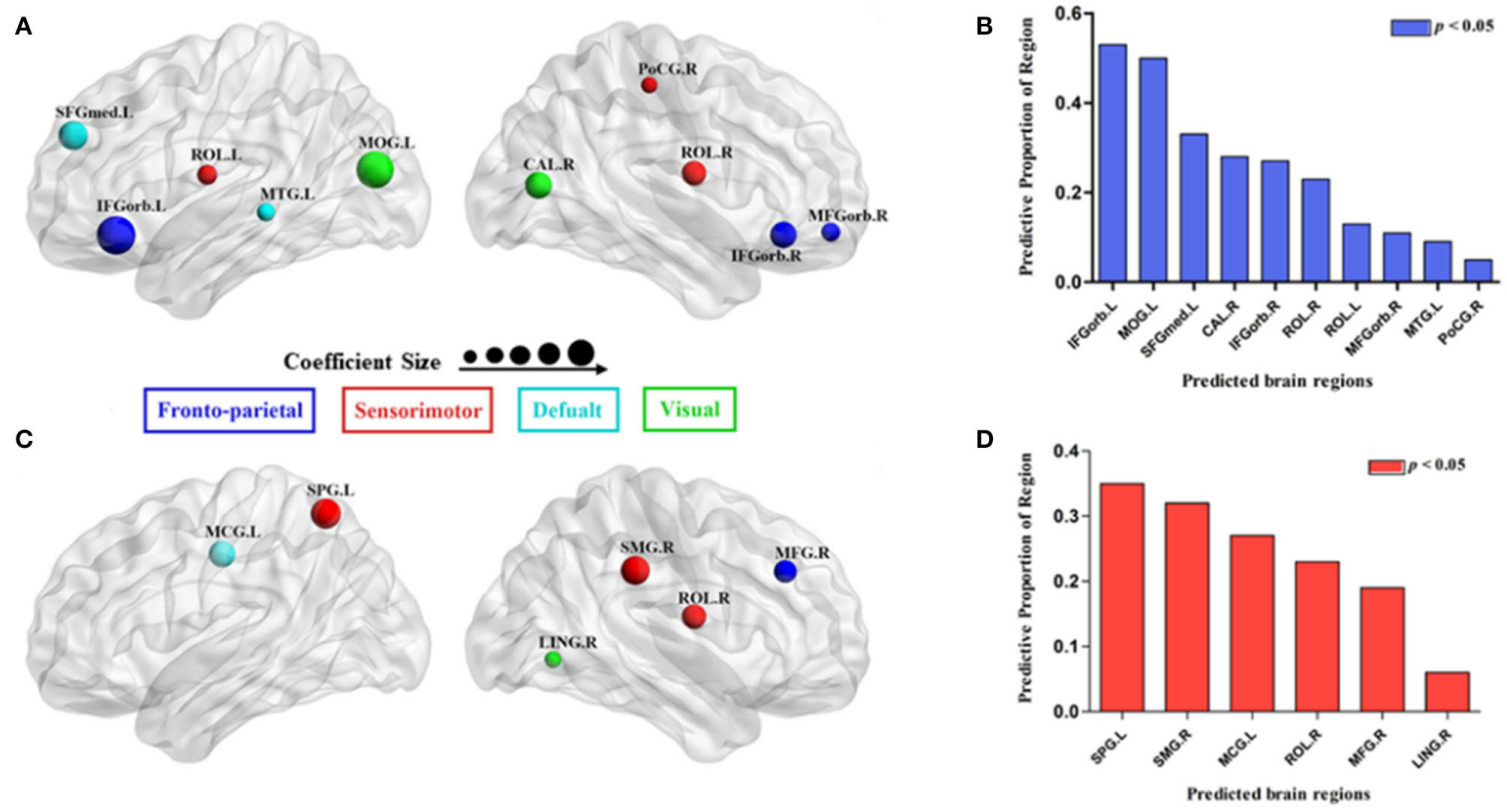
Surface visualization of the brain regions that significantly predicted the integrated network parameters of the creative-network (**A** for integrated local efficiency; **C** for integrated global efficiency); the model related to each region was significant (*p* < 0.05; **B** for integrated local efficiency; **D** for integrated global efficiency). The radius of the node corresponds to the coefficient size. The different colors of the regions indicate different subnetworks.

Using MLRA, we found that the integrated nodal properties of the CTP-network could explain the variance in the integrated network parameters of the creative-network. With respect to the integrated *E*_loc−nodal_, the brain regions associated with the integrated *E*_loc_ of the creative-network (Adjusted *R*^2^ = 0.779, *p* < 0.030) included the left middle temporal gyrus (MTG), right inferior frontal gyrus orbital part (IFGorb), left IFGorb, left MOG, left Rolandic operculum (ROL), left medial superior frontal gyrus (SFGmed), right MFGorb, right postcentral gyrus (PoCG), right ROL, and right calcarine cortex (CAL). In particular, the left IFGorb, left MOG, left SFGmed, right CAL, and right IFGorb predicted a variance of 53, 50, 33, 28, and 27% in the integrated *E*_loc_ of the creative-network (Table 2 and Figures 4A,B).

**Table 2:** Brain locations involved in the multiple linear regression analysis (MLRA) for integrated nodal parameters in the critical time point- (CTP-) network.

**Parameters**	**Regions**	**Hemisphere**	**Module**
**Integrated** ***E**_***loc*−*nodal***_*			
	MTG	L	Default
	IFGorb	R	Fronto-parietal
	IFGorb	L	Fronto-parietal
	MOG	L	Visual
	ROL	L	Sensorimotor
	SFGmed	L	Default
	MFGorb	R	Fronto-parietal
	PoCG	R	Sensorimotor
	ROL	R	Sensorimotor
	CAL	R	Visual
**Integrated** ***E**_***glob*−*nodal***_*			
	MCG	L	Default
	SMG	R	Sensorimotor
	LING	R	Visual
	ROL	R	Sensorimotor
	MFG	R	Fronto-parietal
	SPG	L	Sensorimotor

*MTG, middle temporal gyrus; IFGorb, inferior frontal gyrus orbital part; MOG, middle occipital gyrus; ROL, rolandic operculum; SFGmed, medial superior frontal gyrus; MFGorb, middle frontal gyrus orbital part; PoCG, postcentral gyrus; CAL, calcarine cortex; MCG, middle cingulum gyrus; SMG, supramarginal gyrus; LING, lingual gyrus; MFG, middle frontal gyrus; SPG, superior parietal gyrus; R, the right hemisphere; L, the left hemisphere*.

In addition, the integrated nodal global efficiency of the CTP-network also explained the interindividual variation of the integrated global efficiency of the creative-network (Adjusted *R*^2^ = 0.8560, *p* < 0.0001). In the integrated *E*_glob−nodal_, the associated regions included the left middle cingulum gyrus (MCG), right supramarginal gyrus (SMG), right LING, right ROL, right MFG, and left superior parietal gyrus (SPG). Specifically, the left SPG, right SMG, left MCG, right ROL, and right MFG showed important effects in explaining the network attributes during the creative task and predicted a variance of 35, 32, 27, 23, and 19% in the integrated *E*_glob_ of the creative-network (Table 2 and Figures 4C,D).

Notably, the model based on the integrated *E*_loc−nodal_ (*E*_glob−nodal_) of the CTP-network failed to describe a variance in the integrated *E*_loc_ (*E*_glob_) of the control-network (all *p* > 0.05). In addition, the model based on the integrated *E*_loc−nodal_ (*E*_glob−nodal_) of the RTP-network in which nodes were the same as the model based on the integrated *E*_loc−nodal_ (*E*_glob−nodal_) of the CTP-network, could not explain variations in the integrated *E*_loc_ (*E*_glob_) of the creative-network (all *p* > 0.05).

## Discussion

Our present study explored the relationship between spontaneous activity in the PVA and visual creativity. We found that: (1) the pattern of spontaneous activity in the PVA may associate with mental imagery; (2) in comparison with the RTP-network, the CTP-network showed an increase in global efficiency and a decrease in local efficiency; (3) the integrated regional network properties of the CTP-network could predict the integrated network properties of the creative-network while the RTP-network could not.

### Spontaneous Activity Pattern in the PVA Associated With Mental Imagery

The current study showed that there were intermittent episodes of strikingly increased activity in the PVA at the resting state. An increase in activity at CTPs of the PVA exceeds the statistical definition of the activation used in the fMRI data analysis. This phenomenon indicated that spontaneous activity in the PVA was non-random and might be associated with certain mental processes. In addition, we found that the brain regions associated with spontaneous activity in the PVA could be divided into visual areas, sensorimotor areas, and PFC. Using multivariate decoding techniques, several studies found that the content of mental imagery could be decoded from the early visual areas (e.g., V1 and V2) (Pearson et al., 2015; Dijkstra et al., 2017; Pearson, 2019). Similarly, previous studies showed that the visual areas and sensorimotor areas jointly activated in visual imagery tasks (Kraut et al., 2003; Assaf et al., 2006). In addition, the frontal areas (including the PFC) were activated in the tasks that require forming and manipulating mental images (Pearson, 2019). Thus, our findings are in accordance with the previous studies and might indicate that the brain activity at CTPs of the PVA is associated with mental imagery (Wang et al., 2008; Zhang et al., 2018).

### Characteristics of Brain Networks Preparing for the Upcoming Visual Creative Task

This study characterizes and compares the topological attributes of brain networks between the different conditions (CTPs vs. RTPs and creative vs. control conditions). Our results revealed that all brain networks under the resting-state and task-state exhibited the small-world attributes. The small-world means that this brain network could keep the optimal balance between the functional integration and segregation, and maximizes the transferring efficiency of information at a relatively low cost (Sporns and Honey, 2006).

In addition, compared with the RTP-network, we found that the integrated global efficiency was increased and the integrated local efficiency was decreased in the CTP-network. The *E*_glob_ reflects the efficiency of the whole network in transferring the information among the nodes, and the *E*_loc_ reflects the local information transferring ability (Latora and Marchiori, 2001). Previous research on creativity neuroimaging has indicated that the functional integration of the whole brain is strongly related to creativity. For example, Beaty et al. (2015) reported that more creative individuals showed a significantly increased *E*_glob_ in a network of brain regions associated with creativity. Similarly, our previous study found that the high creativity group showed an increased *E*_glob_ compared with the low creativity group at the resting state (Jiao et al., 2017).

According to the information theory, a network topology facilitates the brain responses to perceptual cues by increasing preparatory resources (Hashmi et al., 2014). Findings of an increase in the integrated global efficiency and a decrease in the local efficiency in the CTP-network may be consistent with the information theory. To our knowledge, spontaneous activity in the PVA is associated with mental imagery (Zhang et al., 2018) and mental imagery is important for visual creativity (Palmiero et al., 2011). Before performing the creative task, the resting-state brain network showed a high degree of functional integration, which might be the one preparing for the upcoming visual creative task.

### Regional Network Parameters of the CTP-Network Predicting the Global Network Parameters of the Creative-Network

We found that the regional network attributes of the CTP-network explained variations in the network efficiency during the creative task while the RTP-network did not. The present findings might suggest that the pattern characteristics of spontaneous activity in the PVA are strongly related to the information processing during the visual creative synthesis task. Previous studies demonstrated that the interregional brain activity correlations were considerably varied over the time (Chang and Glover, 2010), and the information of a specific brain network is centrally stored at CTPs containing the most important statistical properties of the dynamical organization (Liu and Duyn, 2013). Thus, our findings that CTP-network attributes rather than the RTP-network could predict the network efficiency of creative-network, which might further support these notions.

First, the results of the current study indicate that the integrated nodal local efficiency of the CTP-network explains the differences in the integrated *E*_loc_ during the visual creative task. Main predictive brain regions were located in the DMN (left SFGmed), FPN (left and right IFGorb), and visual network (left MOG and right CAL). Previous studies have found the activation of the IFG and SFG in creativity tasks (Benedek et al., 2014; Saggar et al., 2015; Chen et al., 2020), and consistently indicated these regions tightly relate to creativity. In addition, the IFGorb and the SFGmed are related to cognitive control and self-generated thought, respectively; both the cognitive functions contributed to creativity (Kleinmintz et al., 2018, 2019).

Notably, except the DMN and FPN, the integrated *E*_loc−nodal_ of the visual network (e.g., the left MOG and right CAL) under CTPs also predicted variations in the integrated *E*_loc_ based on the visual creative synthesis task. A recent study found that the high visual-spatial creativity associated with a stronger resting-state FC between the visual network, DMN, and FPN (Chen et al., 2019). Similarly, Jiao et al. (2017) reported that the network attributes of the occipital regions could predict the creative ability of individuals. In addition, previous studies have suggested that the visual cortex is involved in the creative idea generation related to the use of visual imagery (Howard-Jones et al., 2005; Chrysikou and Thompson-Schill, 2011). Neuroimaging studies have also found that the generation and manipulation of imagery were important in the visual creative synthesis task (Cai et al., 2017), and spontaneous activity in the PVA was related to mental imagery (Zhang et al., 2018). Therefore, by analysing the relation of spontaneous activity in the PVA to brain activity evoked by the visual creative synthesis task, the present findings might suggest that not only the DMN and FPN but also the visual network were essential for visual creativity.

In addition, we further identified that the integrated nodal global efficiency predicted variations in the integrated *E*_glob_ during the visual creative task. A recent study indicated a strong association between the global efficiency of functional brain networks and the visual creative ability of individuals (Jiao et al., 2017). In addition, the main predictive brain regions were in the sensorimotor network (right SMG, ROL, and left SPG), DMN (left MCG), and FPN (right MFG). A previous study showed that the left parietal cortex was more activated in the visual creative task than in the control task (Aziz-Zadeh et al., 2013). Similarly, Gansler et al. (2011) found that visual creative individuals have higher volume in the SPG and SMG, and these regions were associated with visuospatial processing in visual creativity. The visual creative synthesis task refers to the use of mental imagery to make unexpected discoveries, which involved the sensorimotor network (action planning), DMN (idea generation), and FPN (evaluation process). Taken together, our results might further support a strong relationship between these brain networks and visual creativity.

### Limitations

The present study has several limitations, and future research should address these limitations. This study is mainly and primarily focused on the relationship between the PVA and visual creativity. However, the visual cortex (network) comprises a much larger number of distinct cortical areas (e.g., V1, V2, and V3). Future studies should gather the areas other than PVA to investigate the role of the visual cortex (network) in creative cognition. Moreover, our study was limited to the sample size and only collected the data of 16 subjects. Although there has been a related study that has provided stable results on the same sample size (Cai et al., 2017), future studies should be based on more subjects to increase the reproducibility and reliability of the results.

## Conclusions

In summary, our results revealed that the characteristic of the brain network from the CTPs of spontaneous activity in the PVA might relate to visual creativity. Compared with the RTP-network, the CTP-network showed an increase in global efficiency and a decrease in local efficiency at the resting state, which may be a brain preparation activity for the upcoming visual creative task. In addition, the integrated regional network parameters of the CTP-network could predict the integrated global network parameters of the creative-network while the RTP-network could not. This result might suggest that the most useful and important information regarding spontaneous activity in the PVA is stored at these CTPs. Meanwhile, during spontaneous activity in the PVA, not only the regions of the DMN and FPN but also the visual network regions are strongly related to the visual creative synthesis task-evoked neural responses. Therefore, our findings were an extension of the previous research and provided an insight into the relationship between the visual cortex and visual creativity.

## Data Availability Statement

The datasets for this article are not publicly available because participants from the present study were assured raw data would remain confidential and would not be shared. Requests to access the datasets should be directed to delong.zhang@m.scnu.edu.cn.

## Ethics Statement

The studies involving human participants were reviewed and approved by The Institutional Review Boards at South China Normal University. The patients/participants provided their written informed consent to participate in this study.

## Author Contributions

YW, DZ, and ML conceived the study. YW performed the data analysis. YW, JL, and DZ wrote this paper. YW, ZW, BL, BJ, YH, PZ, HY, RY, and SY collected the MRI data. All authors have approved the final manuscript.

## Conflict of Interest

The authors declare that the research was conducted in the absence of any commercial or financial relationships that could be construed as a potential conflict of interest.

## References

[B1] Assaf M. Calhoun V. D. Kuzu C. H. Kraut M. A. Rivkin P. R. Hart J. Jr.. (2006). Neural correlates of the object-recall process in semantic memory. Psychiatry Res. Neuroimaging 147, 115–126. 10.1016/j.pscychresns.2006.01.002 16938439

[B2] Aziz-Zadeh L. Liew S. L. Dandekar F. (2013). Exploring the neural correlates of visual creativity. Soc. Cogn. Affect. Neurosci. 8, 475–480. 10.1093/scan/nss021 22349801 PMC3624959

[B3] Beaty R. E. Benedek M. Kaufman S. B. Silvia P. J. (2015). Default and executive network coupling supports creative idea production. Sci. Rep. 5:10964. 10.1038/srep10964 26084037 PMC4472024

[B4] Beaty R. E. Benedek M. Silvia P. J. Schacter D. L. (2016). Creative cognition and brain network dynamics. Trends Cogn. Sci. 20, 87–95. 10.1016/j.tics.2015.10.004 26553223 PMC4724474

[B5] Benedek M. Jauk E. Fink A. Koschutnig K. Reishofer G. Ebner F.. (2014). To create or to recall? Neural mechanisms underlying the generation of creative new ideas. Neuroimage 88, 125–133. 10.1016/j.neuroimage.2013.11.021 24269573 PMC3991848

[B6] Boccia M. Piccardi L. Palermo L. Nori R. Palmiero M. (2015). Where do bright ideas occur in our brain? Meta-analytic evidence from neuroimaging studies of domain-specific creativity. Front. Psychol. 6:1195. 10.3389/fpsyg.2015.01195 26322002 PMC4531218

[B7] Bullmore E. T. Suckling J. Overmeyer S. Rabe-Hesketh S. Taylor E. Brammer M. J. (1999). Global, voxel, and cluster tests, by theory and permutation, for a difference between two groups of structural MR images of the brain. IEEE Trans. Med. Imaging 18, 32–42. 10.1109/42.750253 10193695

[B8] Cai Y. Zhang D. Liang B. Wang Z. Li J. Gao Z.. (2017). Relation of visual creative imagery manipulation to resting-state brain oscillations. Brain Imaging Behav. 12, 258–273. 10.1007/s11682-017-9689-8 28271439

[B9] Chang C. Glover G. H. (2010). Time-frequency dynamics of resting-state brain connectivity measured with fMRI. Neuroimage 50, 81–98. 10.1016/j.neuroimage.2009.12.011 20006716 PMC2827259

[B10] Chen Q. Beaty R. E. Cui Z. Sun J. He H. Zhuang K.. (2019). Brain hemispheric involvement in visuospatial and verbal divergent thinking. Neuroimage 15:116065. 10.1016/j.neuroimage.2019.116065 31398434

[B11] Chen Q. Beaty R. E. Qiu J. (2020). Mapping the artistic brain: common and distinct neural activations associated with musical, drawing, and literary creativity. Hum. Brain Mapp. 41, 3403–3419. 10.1002/hbm.25025 32472741 PMC7375056

[B12] Chrysikou E. G. Thompson-Schill S. L. (2011). Dissociable brain states linked to common and creative object use. Hum. Brain Mapp. 32, 665–675. 10.1002/hbm.21056 20533561 PMC3846690

[B13] De Pisapia N. Bacci F. Parrott D. Melcher D. (2016). Brain networks for visual creativity: a functional connectivity study of planning a visual artwork. Sci. Rep. 6:39185. 10.1038/srep39185 27991592 PMC5171814

[B14] Dietrich A. Kanso R. (2010). A review of EEG, ERP, and neuroimaging studies of creativity and insight. Psychol. Bull. 136, 822–848. 10.1037/a0019749 20804237

[B15] Dijkstra N. Bosch S. E. van Gerven M. A. (2017). Vividness of visual imagery depends on the neural overlap with perception in visual areas. J. Neurosci. 37, 1367–1373. 10.1523/JNEUROSCI.3022-16.2016 28073940 PMC6596858

[B16] Finke R. A. (2014). Creative Imagery: Discoveries and Inventions in Visualization. Hillsdale: Psychology Press.

[B17] Finke R. A. Slayton K. (1988). Explorations of creative visual synthesis in mental imagery. Mem. Cogn. 16, 252–257. 10.3758/BF03197758 3393086

[B18] Friston K. J. Williams S. Howard R. Frackowiak R. S. Turner R. (1996). Movement-related effects in fMRI time-series. Magn. Reson. Med. 35, 346–355. 10.1002/mrm.1910350312 8699946

[B19] Gansler D. A. Moore D. W. Susmaras T. M. Jerram M. W. Sousa J. Heilman K. M. (2011). Cortical morphology of visual creativity. Neuropsychologia 49, 2527–2532. 10.1016/j.neuropsychologia.2011.05.001 21600905

[B20] Hashmi J. A. Kong J. Spaeth R. Khan S. Kaptchuk T. J. Gollub R. L. (2014). Functional network architecture predicts psychologically mediated analgesia related to treatment in chronic knee pain patients. J. Neurosci. 34, 3924–3936. 10.1523/JNEUROSCI.3155-13.2014 24623770 PMC3951694

[B21] Howard-Jones P. A. Blakemore S. J. Samuel E. A. Summers I. R. Claxton G. (2005). Semantic divergence and creative story generation: an fMRI investigation. Cogn. Brain Res. 25, 240–250. 10.1016/j.cogbrainres.2005.05.013 15993573

[B22] Huang P. Qiu L. Shen L. Zhang Y. Song Z. Qi Z.. (2013). Evidence for a left-over-right inhibitory mechanism during figural creative thinking in healthy nonartists. Hum. Brain Mapp. 34, 2724–2732. 10.1002/hbm.22093 22522783 PMC6870349

[B23] Jiao B. Zhang D. Liang A. Liang B. Wang Z. Li J.. (2017). Association between resting-state brain network topological organization and creative ability: evidence from a multiple linear regression model. Biol. Psychol. 129, 165–177. 10.1016/j.biopsycho.2017.09.003 28890001

[B24] Kleinmintz O. M. Abecasis D. Tauber A. Geva A. Chistyakov A. V. Kreinin I.. (2018). Participation of the left inferior frontal gyrus in human originality. Brain Struct. Funct. 223, 329–341. 10.1007/s00429-017-1500-5 28828749

[B25] Kleinmintz O. M. Ivancovsky T. Shamay-Tsoory S. G. (2019). The two-fold model of creativity: the neural underpinnings of the generation and evaluation of creative ideas. Curr. Opin. Behav. Sci. 27, 131–138. 10.1016/j.cobeha.2018.11.004

[B26] Kraut M. A. Calhoun V. Pitcock J. A. Cusick C. Hart J. Jr. (2003). Neural hybrid model of semantic object memory: implications from event-related timing using fMRI. J. Int. Neuropsychol. Soc. 9, 1031–1040. 10.1017/S135561770397007X 14738284

[B27] Latora V. Marchiori M. (2001). Efficient behavior of small-world networks. Phys. Rev. Lett. 87:198701. 10.1103/PhysRevLett.87.198701 11690461

[B28] Liu X. Duyn J. H. (2013). Time-varying functional network information extracted from brief instances of spontaneous brain activity. *Proc. Natl. Acad. Sci*. U.S.A. 110, 4392–4397. 10.1073/pnas.1216856110 PMC3600481 23440216

[B29] Palmiero M. Cardi V. Belardinelli M. O. (2011). The role of vividness of visual mental imagery on different dimensions of creativity. Creat. Res. J. 23, 372–375. 10.1080/10400419.2011.621857

[B30] Palmiero M. Nori R. Aloisi V. Ferrara M. Piccardi L. (2015). Domain-specificity of creativity: a study on the relationship between visual creativity and visual mental imagery. Front. Psychol. 6:1870. 10.3389/fpsyg.2015.01870 26648904 PMC4664616

[B31] Park H. R. Kirk I. J. Waldie K. E. (2015). Neural correlates of creative thinking and schizotypy. Neuropsychologia 73, 94–107. 10.1016/j.neuropsychologia.2015.05.007 25979607

[B32] Pearson J. (2019). The human imagination: the cognitive neuroscience of visual mental imagery. Nat. Rev. Neurosci. 20, 624–634. 10.1038/s41583-019-0202-9 31384033

[B33] Pearson J. Naselaris T. Holmes E. A. Kosslyn S. M. (2015). Mental imagery: functional mechanisms and clinical applications. Trends Cogn. Sci. 19, 590–602. 10.1016/j.tics.2015.08.003 26412097 PMC4595480

[B34] Pidgeon L. M. Grealy M. Duffy A. H. Hay L. McTeague C. Vuletic T.. (2016). Functional neuroimaging of visual creativity: a systematic review and meta-analysis. Brain Behav. 6:e00540. 10.1002/brb3.540 27781148 PMC5064346

[B35] Saggar M. Quintin E. M. Kienitz E. Bott N. T. Sun Z. Hong W. C.. (2015). Pictionary-based fMRI paradigm to study the neural correlates of spontaneous improvisation and figural creativity. Sci. Rep. 5:10894. 10.1038/srep10894 26018874 PMC4446895

[B36] Sporns O. Honey C. J. (2006). Small worlds inside big brains. Proc. Natl. Acad. Sci. U.S.A. 103, 19219–19220. 10.1073/pnas.0609523103 17159140 PMC1748207

[B37] Tagliazucchi E. Balenzuela P. Fraiman D. Chialvo D. R. (2012). Criticality in large-scale brain FMRI dynamics unveiled by a novel point process analysis. Front. Physiol. 3:15. 10.3389/fphys.2012.00015 22347863 PMC3274757

[B38] Tijms B. M. Seriès P. Willshaw D. J. Lawrie S. M. (2012). Similarity-based extraction of individual networks from gray matter MRI scans. Cerebral Cortex 22, 1530–1541. 10.1093/cercor/bhr221 21878484

[B39] Wang K. Jiang T. Yu C. Tian L. Li J. Liu Y.. (2008). Spontaneous activity associated with primary visual cortex: a resting-state FMRI study. Cerebral Cortex 18, 697–704. 10.1093/cercor/bhm105 17602140

[B40] Watts D. J. Strogatz S. H. (1998). Collective dynamics of ‘small-world'networks. Nature 393:440. 10.1038/30918 9623998

[B41] Zhang Z. Zhang D. Wang Z. Li J. Lin Y. Chang S.. (2018). Intrinsic neural linkage between primary visual area and default mode network in human brain: evidence from visual mental imagery. Neuroscience 379, 13–21. 10.1016/j.neuroscience.2018.02.033 29524639

[B42] Zhu W. Chen Q. Xia L. Beaty R. E. Yang W. Tian F.. (2017). Common and distinct brain networks underlying verbal and visual creativity. Hum. Brain Mapp. 38, 2094–2111. 10.1002/hbm.23507 28084656 PMC6866727

